# Evidence-Based Neuroethics, Deep Brain Stimulation and Personality - Deflating, but not Bursting, the Bubble

**DOI:** 10.1007/s12152-018-9392-5

**Published:** 2018-12-03

**Authors:** Jonathan Pugh, Laurie Pycroft, Hannah Maslen, Tipu Aziz, Julian Savulescu

**Affiliations:** 1 Department of Philosophy, The University of Oxford, The Oxford Uehiro Centre for Practical Ethics, Oxford, UK; 2 Oxford Functional Neurosurgery, University of Oxford, Oxford, UK

**Keywords:** Deep brain stimulation, Personality, Identity, Self, Autonomy, Agency, Authenticity, Evidence-based medicine, Surgical trials

## Abstract

Gilbert et al. have raised important questions about the empirical grounding of neuroethical analyses of the apparent phenomenon of Deep Brain Stimulation ‘causing’ personality changes. In this paper, we consider how to make neuroethical claims appropriately calibrated to existing evidence, and the role that philosophical neuroethics has to play in this enterprise of ‘evidence-based neuroethics’. In the first half of the paper, we begin by highlighting the challenges we face in investigating changes to PIAAAS following DBS, explaining how different trial designs may be of different degrees of utility, depending on how changes to PIAAAS following DBS are manifested. In particular, we suggest that the trial designs Gilbert et al. call for may not be able to tell us whether or not DBS directly causes changes to personality. However, we suggest that this is not the most significant question about this phenomenon; the most significant question is whether these changes should matter morally, however they are caused. We go on to suggest that neuroethical analyses of novel neuro-interventions should be carried out in accordance with the levels of evidence hierarchy outlined by the Centre for Evidence-Based Medicine (CEBM), and explain different ways in which neuroethical analyses of changes to PIAAAS can be evidence-based on this framework. In the second half of the paper, we explain how philosophical neuroethics can play an important role in contributing to mechanism-based reasoning about potential effects on PIAAAS following DBS, a form of evidence that is also incorporated into the CEBM levels of evidence hierarchy.

Neuroethical analyses of medical therapies must always be responsive to empirical research regarding the clinical realities of those interventions. Accordingly, Gilbert et al.’s paper, which calls attention to the lack of empirical support for the frequently voiced claim that “DBS causes personality changes”, warrants serious attention [1]. We agree with many of the important, though provocative claims in Gilbert et al.’s paper. In particular, we agree that neuroethicists should do more to contextualise their discussions of changes to personality, identity, autonomy, authenticity, agency and self (PIAAAS) following DBS treatment, both to existing data concerning the incidence of such events, and also the evidence of the beneficial effects of DBS for many patients.

However, as one of the many parties to the neuroethical discussion of alterations to PIAAAS following DBS treatment, we shall claim that Gilbert et al.’s arguments may not be as far-reaching for neuroethical discussion as they may first appear. First, we shall highlight some of the challenges of investigating changes to PIAAAS following DBS, explaining how different trial designs may be of different degrees of utility, depending on how changes to PIAAAS following DBS are manifested. In particular, we point out that there may be significant obstacles to carrying out randomized controlled trials of the sort that will be needed to answer some important questions about PIAAAS. We go on to raise doubts about the moral significance of some of these questions, and highlight an appropriate role for case studies and mechanism-based reasoning in this context. Furthermore, we shall sketch out ways in which philosophical neuroethics can have an important role to play in discussions of DBS, and in what we call evidence-based neuroethics more generally. In doing so, we shall also suggest how existing work in this area may be useful in this context, even if it does not reflect evidence from first hand primary studies.

## DBS, PIAAAS, and Evidence-Based Neuroethics

The latter half of the twentieth century saw the evidence-based medicine revolution, in which doctors were enjoined to rely on empirical evidence from high quality clinical trials, rather than more traditional forms of mechanistic reasoning from basic sciences in order to inform their clinical judgment [2]. In many ways, Gilbert et al’s paper can be read as a similar call for neuroethics, to make neuroethical claims appropriately calibrated to existing evidence.

In view of this, perhaps the most striking finding in Gilbert et al.’s paper is that only 12.5% of first-hand primary studies provide significant evidence to support a link between DBS and alterations to PIAAAS [1].^1^ Although neuroethicists also tend to draw on evidence from case reports, Gilbert et al. point out that such reports do not provide robust evidence, given problems with their internal validity. Furthermore, they note that the 8 primary studies investigating this phenomenon all lacked a control group; accordingly, Gilbert et al. suggest that even these more robust sources of evidence, leave open the possibility that the postoperative effects on PIAAAS evident in these studies may be an indirect result of the amelioration of the patient’s condition, rather than a direct result of DBS per se. Accordingly, they suggest that changes to PIAAAS following DBS treatment may in fact be a result of the patient experiencing difficulties with social integration, or of the ‘unmasking’ of extant psychiatric symptoms previously masked by the patient’s other symptoms. Indeed, Gilbert et al. note that this was the interpretation of the authors of two of these first-hand studies [1, 3, 4].

In view of this, one significant conclusion that Gilbert et al. draw is that “.. . the theoretical neuroethics literature may rely on unsubstantiated speculative assumptions in lieu of robust evidence”, and that “no generalizable conclusions and recommendations should be drawn from such limited data”. Accordingly, the call for more robust, systematic qualitative studies to investigate causal effects and the incidence of these postoperative effects [1]. More specifically, they suggest that studies investigating effects on PIAAAS “should differentiate between active and inactive control groups”, due to the different effect estimates that are obtained from these groups.

There are certainly deficiencies in the way that effects on PIAAAS are currently investigated, and there are clearly ways in which it may be possible to study the prevalence of such effects more systematically. This is particularly so in the relatively large population of patients undergoing DBS for movement disorders. Indeed, a significant part of Gilbert et al.’s concern is that we need far more evidence if we are to form an accurate impression of the prevalence of changes to PIAAAS following DBS treatment. We wholeheartedly agree with this point.

However, their comments also suggest that they are also interested in a further question, which we take as our focus in the first half of this paper. Their comments indicate that they are also interested in the question of whether changes to PIAAAS observed amongst patients are directly ‘induced’ by stimulation, or whether they are an indirect effect of stimulation (of the sort that Agid et al. mention as quoted by Gilbert et al. [1] [p.4]). On this understanding, an indirect effect is one that is attributable to the amelioration of the patient’s condition evinced by DBS, but not directly *induced* by DBS.

Call the aforementioned question ‘the causal question’.^2^ Although Gilbert et al. make some broad recommendations about how we might scientifically approach the causal question, we believe that there are limits to what the trial designs they advocate can tell us. We shall now explain why this is the case, and also why we doubt the moral significance of answering this question.

First, we should acknowledge that if there were strong evidence from sham-controlled studies to suggest that participants in the sham arm of such trials experienced alterations to PIAAAS as much as (or more than) those undergoing active DBS, this evidence would help to answer the causal question. Such evidence would suggest that alterations to PIAAAS are attributable to a placebo/nocebo effect,^3^ and that stimulation *itself* is not directly causing these changes. Whilst Gilbert et al. briefly mention that such a result is apparent in Schupbach et al.s’ widely discussed study [4], as far as we are aware this is an isolated result that should be treated with some caution.

Assume though that there are some cases in which the patient’s experience of an alteration to PIAAAS is not attributable to a placebo/nocebo effect. If so, there are two remaining hypotheses:

1. Stimulation is directly causing changes to PIAAAS
2. Changes to PIAAAS are an indirect effect of the amelioration of the patient’s condition evinced by DBS treatment.

Consider how one might try to establish which hypothesis is correct. Gilbert et al. advert to the importance of including both randomized inactive control groups (i.e. participants who do not receive an active comparison treatment) and active control groups (participants who do receive an approved alternative treatment) in future studies. However, as we shall now explain, it is not clear that the inclusion of such groups will always be sufficient to answer the causal question.

For some patients, it may be possible to design trials that can give use evidence that might favour one of the above hypotheses. One appealing feature of DBS is that it allows for within-patient comparisons; as well as assessing patients pre-operatively, patients can also be assessed post-operatively, both on and off stimulation. A trial that included such *within* patient comparisons on and off stimulation might be able to give us evidence that would be relevant to answering the causal question. This would be so if the data were indicative of either of the following two scenarios:

- **Scenario A**
  - In the ‘on’ condition, subjects do *not* receive a therapeutic benefit, but they do undergo changes to PIAAAS.
    And
  - When stimulation is off, changes are absent.
- **Scenario B**
  - In the ‘on’ condition, subjects receive a therapeutic benefit, but they do *not* undergo changes to PIAAAS.
    And
  - In the ‘off’ condition, changes are absent.

Scenario A would provide evidence for hypothesis [1], that stimulation is directly causing changes to PIAAAS. Scenario B would provide evidence for the claim that the amelioration of the patient’s condition does not entail changes to PIAAAS, weakening the evidential basis for hypothesis [2].

Other trial designs might also provide useful information about hypothesis [1]. This hypothesis can be divided into two sub-hypotheses: (i) Stimulation of the *intended* neural target causes PIAAAS changes directly or (ii): Stimulation of *unintended* neural structures, which may be unavoidable when delivering effective therapeutic relief due to the proximity of the targeted region, results in changes to PIAAAS. It might be possible to test whether 1(i) or 1(ii) is correct by investigating whether changes to stimulation parameters differentially affect therapeutic efficacy and PIAAAS disturbance.

If hypothesis 1(i) were true, we would expect to see PIAAAS disturbances even in patients who are receiving DBS that is optimal in terms of electrode placement and stimulation settings. Alterations to stimulation settings would likely cause changes in symptom relief and PIAAAS that correlate well. Conversely, if hypothesis 1(ii) were true, we would expect greater independence between changes in therapeutic efficacy and PIAAAS when stimulation parameters are manipulated, as different neural structures would be affected to greater or lesser degrees depending on their adjacency to the electrode, and the intensity of stimulation being received. Emerging technological developments in DBS hardware are making such experiments more feasible, particularly segmented electrode designs, which allow current to be “steered” in a given direction. This allows for subtle alteration of the volume of neural tissue being activated and could, therefore, facilitate investigation of the physiological and anatomical correlates of putative PIAAAS changes by allowing researchers to better distinguish between side-effects arising from stimulation of the targeted structure, versus off-target effects.

However, the problem is that there are likely to be a considerable number of patients who undergo changes to PIAAAS in which these designs can tell us little about whether we should favour hypothesis 1 over hypothesis 2. To see why, suppose data from a within patient comparison on/off trial indicated the following:

- **Scenario C**
  - In the ‘on’ condition, subjects receive a therapeutic benefit, but they also undergo changes to PIAAAS.
    And
  - In the ‘off’ condition, changes to PIAAAS are absent.

In such a scenario, it will be very difficult to answer the causal question; the data is consistent with both the hypothesis that stimulation directly causes changes to PIAAAS *and* the hypothesis that such changes are a result of the amelioration of the patient’s condition. Crucially, existing empirical evidence of changes to PIAAAS suggest that such change often occur in tandem with the therapeutic benefits of DBS in the manner that scenario C outlines. Indeed, this is reflected in the title of Agid’s seminal patient interview study of DBS patients, which notes that in DBS it can be the case that the ‘doctor is happy, but the patient less so’ [3].

If there were a considerable number of patients who fit scenario C in a within patient, on/off trial design, then in order to isolate the relevant variables in way that can answer the causal question, we would need a different design. One would need a design including a randomized control group whose members had *not* received active DBS, but whose condition had been ameliorated to the same extent (and from the same baseline) as the group receiving active DBS. In this design, if members of the group receiving DBS experienced alterations to PIAAAS but the active control group did not, then this would provide evidence that DBS is directly causing these alterations, rather than the mere amelioration of the subject’s condition. Simply comparing the active DBS group to randomized active and inactive control groups cannot furnish us with such evidence, unless the amelioration of the member’s conditions is held constant across groups.

This is problematic because it is difficult to imagine circumstances in which it would be feasible to recruit such a control group. There are of course the usual practical obstacles to typical clinical trial recruitment (mentioned by Gilbert et al. in a footnote [1]). More significantly though, since DBS is often used as a last resort treatment after other interventions have failed (particularly in the psychiatric context) there may be few patients whose condition could be ameliorated by an alternative intervention to the same extent (and from the same baseline) as patients receiving active DBS, and who could thus constitute an adequate active control group. Indeed, even if there were such patients, it may be ethically dubious to perform such a trial just to investigate the causal question in this way.^4^ After all, DBS is a highly invasive procedure with perioperative and postoperative physiological risks; accordingly, assuming that permissible clinical trials should (i) minimize harms to research participants and (ii) investigate clinically important goals, it would only be permissible to carry out such a trial if the comparably efficacious intervention had a broadly similar risk profile to DBS, and if answering the causal question was clinically important.

Part of the reason that we believe such research would be ethically dubious is that the clinical importance of the causal question is questionable. Whatever the answer to the causal question is, we can surely agree that the phenomenon of perceived changes to PIAAAS are apparent in the literature, albeit not to the extent that is proportionate to the attention the phenomenon has received in the theoretical neuroethics literature. In our view, this phenomenon is of clinical significance regardless of whether it is brought about directly by DBS or as an indirect result of the patient’s amelioration. Indeed, focusing too much on the causal question in DBS may risk downplaying or overlooking ways in which other neurological interventions may indirectly affect PIAAAS.

The answer to the causal question might have a great deal of clinical significance if there were other alternative treatments for patients who were suitable candidates for DBS, which had comparable safety and efficacy profiles to DBS, but that did not pose a threat of directly altering PIAAAS. In such circumstances, establishing the answer to the causal question regarding DBS would be important for making an all-things-considered comparison between DBS and such alternative treatment modalities with regard to the patient’s best interests. However, DBS is typically used as a last resort treatment; suitable candidates for DBS typically do not have alternative effective treatment options.^5^

It might be argued that ablative neurosurgical interventions could represent a potential alternative treatment for candidates for DBS treatment. [8] Perhaps patients undergoing ablative neurosurgery might even serve as the sort of control group that could help answer the casual question outlined above. However, unless it was possible to enroll a sufficient number of participants who were unsuitable candidates for DBS, such a study would, we believe, fail the criterion of clinical equipoise. In the context of Parkinson’s Disease, evidence has established that DBS has fewer adverse effects and results in a greater overall improvement in function [9, 10]. In the context of psychiatric DBS, there is admittedly a lack of such comparative evidence, and the risk profiles of ablative neurosurgery and DBS share some broad similarities. Nonetheless, we believe that the relatively reversible nature of DBS means that DBS poses significantly lower risk of all things considered harm to patients than ablative neurosurgery, the effects of which are largely irreversible. In particular, as some of us have argued with other colleagues elsewhere, this feature may make DBS a preferable treatment modality for some psychiatric disorders where the patient’s perception of control plays a central role in the psychopathology [11].

It might be argued that by downplaying the significance of the causal question, we are conceding that DBS does not raise any particularly novel ethical questions. If what matters ethically are the alterations to PIAAAS per se, rather than alterations that are directly caused by DBS, then it might seem that our moral concerns about DBS in this regard are just the same concerns that we should have about *any* medical treatment that might radically ameliorate a patient’s medical condition, since they too might plausibly lead to alterations to PIAAAS. After all, there is no reason to believe that other such interventions are immune to the problem of whether observed personality effects are caused by the intervention itself or by changes in the patient’s underlying condition. Perhaps it might be argued that our claims here serve to burst the bubble of DBS as a topic worthy of specific neuroethical attention.

However, we believe that this conclusion should be resisted, due to particular features of DBS that distinguish it from other medical interventions that might potentially lead to such changes, features that raise important moral questions. First, the reversibility of DBS means that DBS raises a set of ethical questions that are distinct from those that are raised by comparable irreversible procedures such as ablative neurosurgery.

An important feature of DBS is that patients must choose if and how to sustain DBS treatment over time. Accordingly, for patients undergoing DBS treatment, there will be multiple points at which the patient’s consent to treatment will need to be solicited, and capacity assessed [11, 12]. In this regard, DBS is importantly different from ablative forms of neurosurgery that might be considered as alternatives to DBS treatment for some patients; ablative neurosurgery is a one-off, irreversible procedure, for which consent need only be solicited prior to the procedure. In the case of DBS, medical teams may face a decision about what to do if is a conflict between the patient’s long term wishes in the absence of stimulation, and their wishes having undergone stimulation, and having potentially been subject to changes to PIAAAS [13].

Yet DBS also differs from other *reversible* direct interventions, like pharmaceuticals. First, DBS allows for more precise targeting of neuronal activation than pharmaceutical interventions. This is important both in terms of spatial precision (targeting a cubic millimetre of brain tissue as opposed to affecting receptor activity in every neuron with a certain drug) and variability; whilst drug dosage may affect the concentration of given agonists/antagonists at the target receptor, in DBS one can alter the volume of tissue being affected and the characteristics of the stimulation being delivered. Crucially, this allows clinical teams to exercise more fine-grained control over the effects of treatment over time, and to respond to physiological, behavioural, and environmental changes over the course of treatment.

Accordingly, questions about which particular neural mechanisms should be targeted are more salient in the context of DBS. Furthermore, unlike pharmaceutical treatments, the effects of DBS are often continuous, and any decision to reduce or *cease* DBS treatment must be active. In contrast, the effects of pharmaceuticals wax and wane as the drug is metabolized, and the decision to *continue* with most pharmaceutical treatments is actively, even if habitually, made every day, often when the effects of the drug are reduced or absent. Thus, there are differences that relate to 1) the range of evaluative standpoints available to the patient regarding the effects of the treatment (whether they have regular epistemic access to what things are like for them off (or less ‘on’) the treatment, including any effects on PIAAAS) and 2) the default therapeutic state in which the patient makes decisions regarding their treatment. If DBS patients are in a state of receiving treatment when they make decisions about continuing treatment, they might potentially be influenced by any changes to PIAAAS.

We hope that the above reflections may serve as a starting point for serious empirical investigation of the PIAAAS issue. To conclude this part of our analysis, what should evidence-based neuroethics look like in context of DBS at present? We agree with Gilbert et al. that the available evidence suggests that changes to PIAAAS following DBS are relatively rare effects that have perhaps been so widely analysed by neuroethicists because of the complexity of the philosophical questions they raise, rather than their prevalence in the clinic. However, the causal question about whether DBS directly causes the changes that *are* evident in some cases is not the most significant question about this phenomenon. The real question is the extent to which (and why) effects on PIAAAS (whether evinced directly by DBS or not) should matter morally. There is, we believe, more work to be done on this conceptual issue; but this work should be grounded by an understanding of the way in which the phenomenon is actually manifested in the clinic. To be sure, if there are alternative treatments that have the same beneficial effects on medical conditions as DBS, but potentially fewer side effects on personality, these should be compared. But such a wealth of options seldom exists.

Accordingly, even those who endorse evidence-based neuroethics may concede that neuroethicists considering DBS may have to rely on lower grades of empirical evidence than randomized controlled trials in the foreseeable future. Yet this does not mean that neuroethical analysis cannot be evidence-based. We believe that neuroethical analyses of the effects of neurointerventions should attend to the relevant levels of evidence hierarchies concerning the nature and prevalence of effects of medical treatments, outlined by the Centre for Evidence-Based Medicine (Table 1).

**Table 1:** Oxford centre for evidence-based medicine levels of evidence hierarchy (Abridged)

Question	Step 1 (Level 1*)	Step 2 (Level 2*)	Step 3 (Level 3*)	Step 4 (Level 4*)	Step 5 (Level 5*)
How common is the problem?	Local and current random sample surveys (or censuses)	Systematic review of surveys that allow matching to local circumstances**	Local non-random sample**	Case-series**	n/a
Does this intervention help? (Treatment Benefits)	Systematic review of randomized trials or n-of-1 trials	Randomized trial or observational study with dramatic effect	Non-randomized controlled cohort/follow-up study**	Case-series, case-control studies, or historically controlled studies**	Mechanism-based reasoning
What are the COMMON harms? (Treatment Harms)	Systematic review of randomized trials, systematic review of nested case-control studies, *n* of-1 trial with the patient you are raising the question about, or observational study with dramatic effect	Individual randomized trial or (exceptionally) observational study with dramatic effect	Non-randomized controlled cohort/follow-up study (post-marketing surveillance) provided there are sufficient numbers to rule out a common harm. (For long-term harms the duration of follow-up must be sufficient.)**	Case-series, case-control, or historically controlled studies**	Mechanism-based reasoning
What are the RARE harms? (Treatment Harms)	Systematic review of randomized trials or n-of-1 trial	Randomized trial or (exceptionally) observational study with dramatic effect		Case-series, case-control, or historically controlled studies**	Mechanism-based reasoning

Oxford Centre for Evidence-Based Medicine Working Group, ‘The Oxford 2011 Levels of Evidence’ [14], http://www.cebm.net/index.aspx?o=5653

*Level may be graded down on the basis of study quality, imprecision, indirectness (study PICO does not match questions PICO), because of inconsistency between studies, or because the absolute effect size is very small; Level may be graded up if there is a large or very large effect size

**As always, a systematic review is generally better than an individual study

Notably, on this hierarchical approach, case reports are not the highest level of evidence conceptually speaking. However, this does not mean that they cannot provide a starting point for neuroethical analysis. In the absence of higher levels of evidence, we believe that neuroethicists should still cite appropriately related relevant empirical evidence, whilst being appropriately constrained by the limitations of the level of evidence presented.

Just as significantly though, there are also ways in which neuroethicists can usefully contribute beyond interpreting extant empirical results. As we shall explain in the following section, neuroethicists may have a role to play in contributing to *mechanism-based reasoning* about potential effects on PIAAAS, a form of evidence that is also incorporated into the CEBM levels of evidence hierarchy.

## PIAAAS and the Role of Philosophical Neuroethics

In their analysis of the theoretical neuroethics literature, Gilbert et al. list a number of papers that they suggest make philosophical speculations about the putative impacts of DBS on PIAAAS, but which “appear not to accurately reflect the conclusions made by the first hand primary studies” [1]. The authors raise the concern that such speculations may have the adverse consequence of dissuading prospective patients from undergoing DBS. This list included a paper discussing the ethics of DBS in the treatment of anorexia nervosa authored by some of the authors of the present article [13]. In the second part of this response, we want to defend some of the claims about authenticity in this paper (and others). We do not do so in a spirit of opposition to Gilbert et al.’s analysis; after all, they explicitly point out that “purely theoretical work is warranted and highly valuable” (p.7) [1] . Rather, in doing so we wish to further develop this position by sketching out the sort of role that philosophical neuroethics should play in discussions of emerging neurotechnologies.

It is quite true that the philosophical analysis of our earlier paper was carried out without being grounded by evidence that patients undergoing DBS for anorexia nervosa had experienced changes to PIAAAS. However, the simple reason for this is that at the time of publication, there was no first hand evidence to draw on. Three years later, DBS for anorexia nervosa remains a highly experimental procedure with only one published 1 year follow-up trial at the time of writing [15]^6^. There are also still no published first hand primary qualitative studies relating patients’ experience of treatment. Furthermore, we did not base our analysis on extrapolations from existing primary studies investigating DBS in Parkinson’s Disease, a strategy that Gilbert et al criticise in their paper [1]. Indeed, given the different stimulation targets and disease population, questions pertaining to PIAAAS in anorexic patients undergoing DBS are quite separate from questions pertaining to PIAAAS in patients undergoing DBS for Parkinson’s Disease.

Instead, we aimed to discuss the potential for alterations to PIAAAS in this context by engaging in philosophically and empirically-based mechanistic reasoning. For this reason, despite the fact that the analysis of our paper lacked direct empirical grounding at the time, we stand by our claim that DBS treatment for anorexia can have implications for authenticity. The plausibility of this claim is grounded by our philosophical analysis of the potential mechanisms that might be employed by DBS in the treatment of anorexia nervosa, analysis that draws on different kinds of empirical research into the disease models of AN, and the potential effects of stimulation. Indeed, a central part of our analysis is that the implications of DBS for autonomy and authenticity depend largely on the mechanism employed.

To illustrate with two examples from that paper, suppose one endorses a disease model of anorexia nervosa according to which anorexia nervosa involves aberrant control over compulsive wants that are characteristic of the disease. On this model, one might view the cortico-striatal thalamic circuit as a potential target for DBS treatment, in so far as evidence suggests that this circuit is implicated in compulsive behaviour [17]. If stimulation of this area served to increase top-down control over compulsive behaviour, then it seems that stimulation need not raise particular concerns about threats to autonomy and/or authenticity [13].

However, suppose instead that one adopts a disease model of anorexia nervosa, according to which it is predominantly a disorder of emotional processing. On this model, it might be claimed that stimulation should target brain areas associated with the modulation of emotional states, such as the subcallosal cingulate, which has been targeted in Lipsman et al.’s study [15]. In contrast to the use of DBS to promote top-down control, it seems plausible to suggest that using DBS to reduce aversive affect at least raises the possibility that the intended effects of stimulation may have implications for the patient’s experience of authenticity. Indeed, there has been a great deal of debate in the recent history of neuroethics about the implications that pharmaceutical targeting affective states might have for authenticity [18, 19].

We also considered the Nucleus Accumbens as a possible target of DBS for anorexia nervosa, under a disease model according to which anorexia nervosa involves aberrant reward processing. In our discussion, we noted that this mechanism could potentially raise some issues for authenticity and/or autonomy, particularly if stimulation served to divorce the patient’s perceived rewardingness of food from their evaluative goals [13]. Interestingly, Sanneke de Haan and colleagues have now published a primary study of patient attitudes amongst individuals who had undergone DBS of the Nucleus Accumbens for the treatment of a different psychiatric disorder, Obsessive Compulsive Disorder [20]. Whilst recognising the difficulties of drawing comparisons across different patient populations, the findings of this study are broadly compatible with both our initial analysis of the potential implications of DBS for patient authenticity on this model, and our later theoretical work.

De Haan et al. explicitly claim that their findings in this study ‘confirm the relevance of authenticity’ to discussions of DBS treatment, since participants responses revealed that they were “particularly concerned about whether they had become more or less themselves following DBS treatment” [20]. They also note that considerations of authenticity are particularly complex in OCD due to the fact that the very aim of medical intervention in psychiatry is to change the recipient’s thinking patterns. As such, it is perhaps unsurprising that the findings of De Haan et al.’s study were mixed; whilst some patients believed that DBS had helped them to become ‘more themselves’, others believed that the treatment had induced specific alien behavioural changes, whilst one respondent believed that DBS had led to more global behavioral changes [20].

These findings are compatible with the broad conclusion that we drew in our original theoretical analysis of stimulation of the same site in anorexia nervosa; in the case of using DBS in the treatment of psychiatric disorders, there is not going to be a straightforward answer to the question of whether DBS facilitates or impedes authenticity. Whilst our analysis focused on the fact that different targeted neural mechanisms could have different implications for autonomy and authenticity, De Haan et al.’s study illustrates the further point that the effect of DBS treatment on authenticity can be patient-specific, even amongst populations who have received stimulation in the same area for the same disorder. Moreover, patients may differ in the strategies they use to assess the authenticity of changes they have undergone, and their own relationship to their disorder [20].

Finally, it is striking that de Haan et al.’s data reflects some of the key themes of the dual-basis framework of authenticity that we outlined in follow-up work to our original analysis [21]. In their discussion, De Haan et al. draw a distinction between (i) DBS causing a patient to become a different person and (ii) DBS causing a patient to become a more open, expressive, or impulsive version of herself. They note that their findings are indicative of the latter rather than the former; patients undergo important changes, but these do not render them discontinuous from earlier versions of themselves. In particular, De Haan et al note that whilst a number of patients felt alienated from certain new behavioural traits, DBS treatment did not seem to change their more fundamental evaluative stance. On the basis of these findings, De Haan et al. endorse a dynamic model of the self, according to which ‘the self’ is understood largely in terms of a dynamical process rather than as a fixed entity. However, they claim that there are important limits to this dynamic process, and that continuous change must be grounded by some fixed elements of the self: as they put it, “not everything goes”. [20]

This echoes key features of the dual-basis framework of authenticity that we have developed in considering DBS in the treatment of anorexia nervosa [21]. On this framework, whilst authenticity should be understood to incorporate significant elements of self-creation and to allow for radical change, the extent of this self-creation can only ever be partial if it is to remain authentic. Authentic, radical change must be performed within the limits of the retention of certain persisting values, characteristics and traits through which we can render our own projects of self-creation as intelligibly part of our own development. In this respect, there is considerable overlap between our proposed framework, and the dynamic model that de Haan et al. develop on the basis of their empirical data.

Moreover, in discussing our dual basis framework, we explained the importance of distinguishing inauthentic behavioural traits from inauthentic values, arguing that treatment affecting the latter would be more problematic than the former. Whilst inauthentic behavioural traits may (but need not) be detrimental to well-being, treatment that evinces inauthentic values raises the question of whether we should respect future decisions (particularly treatment decisions) that are grounded by those values. Interestingly, de Haan et al.’s model implicitly reflects this theoretical distinction; although some patients reported that they felt alienated from certain new behavioural traits, they did not believe that treatment changed their values. Our point here is that theoretical neuroethical discussions had not only delineated the possibility of this distinction; it also provided an explanation of its moral significance.

We are not claiming that De Haan’s study proves (or disproves) the conclusions of our theoretical analyses of authenticity in the context of DBS and anorexia nervosa. Indeed, there are some important differences between De Haan et al.’s discussion of the implications of their findings, and the framework outlined in the aforementioned works. Moreover, de Haan’s data deals with a specific patient population and stimulation of a specific neural target that differs from those considered in our more abstract theoretical discussions. However, in light of the above, it is perhaps somewhat uncharitable to suggest that our earlier work is problematic because “it does not accurately reflect primary studies”. Not only was this analysis performed prior to the existence of any directly relevant primary studies, the most relevant primary study that has emerged following the publication of our discussions of authenticity in the context of psychiatric DBS reflect a number of the key themes that were developed our work. We should thus welcome the fact that risks to authenticity have now been acknowledged as a potential harm in published clinical ethics guidelines for Deep Brain Stimulation in the treatment of anorexia nervosa [22].

Instead, we believe that our earlier work is an example of an important role that philosophical neuroethics can play in evidence-based neuroethics. DBS is now an established treatment for movement disorders, and we agree with Gilbert et al. to the extent that philosophical neuroethics on PIAAAS should now be grounded by the robust empirical research of the sort that the Gilbert et al. describe in their paper. However, DBS is increasingly being considered as an experimental treatment across a range of disorders, including psychiatric disorders. Not only are there very few primary studies of patient attitudes towards such interventions, there is also a lack of consensus about the most appropriate neural target for many of these disorders. Accordingly, there is a range of different neural mechanisms that might be targeted by DBS, for a range of different patient populations.

As Gilbert et al. rightly point out, we cannot simply assume that alterations to PIAAAS that are (occasionally) observed amongst one population of patients, being stimulated at one neural target, will translate straightforwardly to another population being stimulated at another target, to treat a different disorder. However, that does not mean that neuroethicists must wait for the data to come in before they can have anything *clinically useful* to say; such work need not just be valuable in the ‘purely theoretical’ sense that Gilbert et al. describe. By carefully attending to existing empirical work about the role of the neural networks being targeted, the role these networks play in the (psycho)pathology under consideration, and features of human psychology, neuroethicists can usefully contribute to mechanism-based reasoning about potential implications for PIAAAS in this context. Further, by bringing models of the self to bear on the potential for DBS treatment leading to alterations to PIAAAS, neuroethicists can help to elucidate the importance of such changes, and why such changes might matter for a particular patient population. Such work need not be scaremongering in the manner that Gilbert et al. rightly caution against; indeed, nuanced analysis may be required to refute the simplistic assumption that any and all interventions into the brain raise concerns about changes to PIAAAS.

Moreover, whilst neuroethicists should of course be responsive to emerging empirical evidence in this regard, a grasp of different models of the self may be essential for fully understanding the implications of what patients are telling us about changes to PIAAAS, and for developing sensitive new tools to capture such changes in the clinic. For instance, authenticity is a philosophical concept not a clinical category. If there is no discussion of it, then it is quite likely that it will not figure in relevant outcome measures of neurointerventions. Neuroethicists should be involved to specify what matters, and what science and medicine should be aiming to measure. Accordingly, whilst we champion evidence-based neuroethics, we also believe that the evidence base in this particular context must be grounded in good neuroethics. We believe that the most clinically useful empirical work on patient understanding of alterations to PIAAAS following DBS treatment should be grounded by a strong philosophical understanding of the central concepts involved in PIAAAS. This is now a welcome emerging trend in the literature as evidenced by De Haan et al. and Gilbert and colleagues own exemplary work in this regard [20, 23].

Concerns about alterations to PIAAAS should be taken in due proportion; they are relatively rare unintended side-effects in non-psychiatric contexts, and DBS can be a hugely beneficial treatment for certain patients. However, alterations to PIAAAS are not just one risk factor amongst many; such changes threaten the most fundamental aspects of how we exist in the world and what we value. That said, there are also no straightforward answers to the question of what implications such changes have for patient well-being and autonomy, and the validity of patient treatment decisions pre and post-stimulation. Indeed, considering this phenomenon in DBS patients can teach neuroethicists a great deal about the theoretical concepts of well-being and autonomy that are operative in these questions. Accordingly, whilst we agree with Gilbert et al. that there may be some scope for deflating the PIAAAS bubble, there are good reasons for thinking that it will not, and we believe should not, be completely burst.

## Footnotes

1 Gilbert et al. define a primary study in this context as “a new clinical report involving at least one patient undergoing psychometric tests or being consulted in a clinical setting or being interviewed for the purpose of examining potential DBS-induced PIAAAS”

2 The distinction that we follow Agid in drawing between direct and indirect causation here should not be confused with the distinction between direct and indirect brain interventions that is sometimes drawn in neuroethics [5]. On the latter distinction, direct interventions change the subject’s thought patterns and behavior by virtue of altering brain structure and function, whilst indirect interventions (such as CBT) seek to change the subject’s thought patterns and behavior, and alter the subject’s brain structure *as result* of these changes. This distinction might overlap to some degree with Agid’s distinction between changes to PIAAAS being caused directly or indirectly by DBS. However, it differs in one crucial respect. Unlike Focquaert and Schermer’s indirect interventions, it seems plausible that the patient may still be passive with respect to changes to PIAAAS that have only been caused by DBS in an indirect sense. This is significant, because one of the most plausible bases for claiming that there is a moral difference between direct and indirect intervention in Focquaert and Schermer’s sense is that their distinction between different kinds of intervention tracks a morally relevant distinction between interventions that require active involvement from the subject, and those where the agent is passive with respect to the changes that the intervention evinces. The crucial point though is that Agid’s direct/indirect causation distinction need not track the active/passive distinction in the same way. We thank an anonymous reviewer for drawing our attention to this possible source of confusion.

3 Whether changes to PIAAAS would qualify as a placebo or a nocebo effect depends on the valence of these changes. We leave open the possibility that changes to PIAAAS might be viewed positively. See [6]

4 As a reviewer observes, we could feasibly perform a trial in which we simply asked patients who had undergone changes to PIAAAS following DBS whether or not they thought that DBS was causally responsible. Whilst we agree that such a trial would be possible, it would provide only an extremely weak evidence base for an answer to the causal question. The reason for this is that we have little reason to suppose that subjects’ own views on the matter should be decisive with regard to establishing a causal mechanism. Indeed, we would not think that this would be a good evidential basis for establishing a causal mechanism between a medical intervention and more traditional kinds of therapeutic effects; by way of analogy, the fact that a trial participant might believe that a drug they have taken is effective is not evidence that the drug they have taken is not a placebo.

5 In the context of Parkinson’s Disease this may be changing, as there is emerging evidence speaking in favour of initiating DBS earlier in the treatment trajectory for some patients [7] . Naturally, if this starts becoming widespread policy, the causal question will increase in clinical relevance, since DBS will be one treatment alternative amongst others at early stages of the disease.

6 However, other trial protocols have been published. See [16]
